# Evaluation of the Antibacterial Potential of Two Short Linear Peptides YI12 and FK13 against Multidrug-Resistant Bacteria

**DOI:** 10.3390/pathogens13090797

**Published:** 2024-09-14

**Authors:** Jingyi Sun, Pan Kong, Jingru Shi, Yuan Liu

**Affiliations:** 1 Jiangsu Co-Innovation Center for Prevention and Control of Important Animal Infectious Diseases and Zoonoses, College of Veterinary Medicine, Yangzhou University, Yangzhou 225009, China; 2 Joint International Research Laboratory of Agriculture and Agri-Product Safety, The Ministry of Education of China, Yangzhou University, Yangzhou 225009, China; 3 Institute of Comparative Medicine, Yangzhou University, Yangzhou 225009, China

**Keywords:** antimicrobial peptide, antibiotic resistance, antibacterial activity, membrane damage

## Abstract

The accelerating spread of antibiotic resistance has significantly weakened the clinical efficacy of existing antibiotics, posing a severe threat to public health. There is an urgent need to develop novel antimicrobial alternatives that can bypass the mechanisms of antibiotic resistance and effectively kill multidrug-resistant (MDR) pathogens. Antimicrobial peptides (AMPs) are one of the most promising candidates to treat MDR pathogenic infections since they display broad-spectrum antimicrobial activities and are less prone to achieve drug resistance. In this study, we investigated the antibacterial capability and mechanisms of two machine learning-driven linear peptide compounds termed YI12 and FK13. We reveal that YI12 and FK13 exhibit broad-spectrum antibacterial properties against clinically significant bacterial pathogens, inducing no or minimal hemolysis in mammalian red blood cells. We further ascertain that YI12 and FK13 are resilient to heat and acid-base conditions, and exhibit susceptibility to hydrolytic enzymes and divalent cations under physiological conditions. Initial mechanistic investigations reveal that YI12 and FK13 compromise bacterial membrane integrity, leading to membrane potential dissipation and excessive reactive oxygen species (ROS) generation. Collectively, our findings highlight the prospective utility of these two cationic amphiphilic peptides as broad-spectrum antibacterial agents.

## 1. Introduction

In the past decades, antibiotics have made substantial contributions to global human health, preserving numerous lives [1]. Nevertheless, the efficacy of conventional antimicrobial medications has been progressively constrained due to the emergence and dissemination of antibiotic resistance. A Report from Lancet has demonstrated that in 2019, 1.27 million deaths were directly caused by drug-resistant bacterial infections [2]. However, few antibiotics have been approved in the past 10 years after development and clinical trials owing to numerous costs and meager successful rates [3,4]. In this case, alternative strategies that can bypass bacterial resistance and effectively kill multidrug-resistant (MDR) pathogens are especially urgently needed.

Antimicrobial peptides (AMPs), also known as host defense peptides (HDPs), are small molecules of peptides between 12 and 50 amino acids existing among all life classes [5]. They belong to the innate immune system and have been reported to be helpful in resisting the invasion of foreign microorganisms [6,7]. Most AMPs present broad-spectrum activities to bacteria, fungi, and viruses [8]. Such far-ranging effects of AMPs are majorly attributed to their versatile modes of action, with external proteins, outer surface lipids, outer membrane proteins (Gram-negative), inner membrane, integral membrane proteins, nucleic acids, and intracellular proteins as potential targets [9,10]. Because of their unique structures and various functions, AMPs not only possess promising antimicrobial activities but also display no cross-resistance with existing antibacterial medicines [11]. Therefore, AMPs have become attractive candidates in the drug-resistance era [12,13], and some, such as daptomycin, have already been used in clinical trials [14].

Although natural AMPs showcase significant bioactivity, they need to overcome the shortcomings of low bioavailability, low target specificity, inducing hemolysis, stimulating cytotoxicity, etc. before final clinical approval [15]. Fortunately, with the aid of modern computational algorithms and artificial intelligence, we can modify natural AMPs through amino acid replacement, subtraction, or even de novo design to perform high optimization [16]. In 2021, Das et al. synthesized 20 brand-new candidates using an in silico screening method accompanied by deep-learning classifiers, discovering two short-length peptides, YK12 and FK13 [4]. Nevertheless, the antibacterial spectrum, safety, stability, and mechanisms of these two peptide candidates remain unclear. In this study, we systematically investigated the physicochemical properties, antibacterial activity, hemolytic activity, and stability of two peptides, and preliminarily explored the underlying modes of action.

## 2. Results

### 2.1. YI12 and FK13 Display Notable Broad-Spectrum Antibacterial Activities

**Figure 1:**
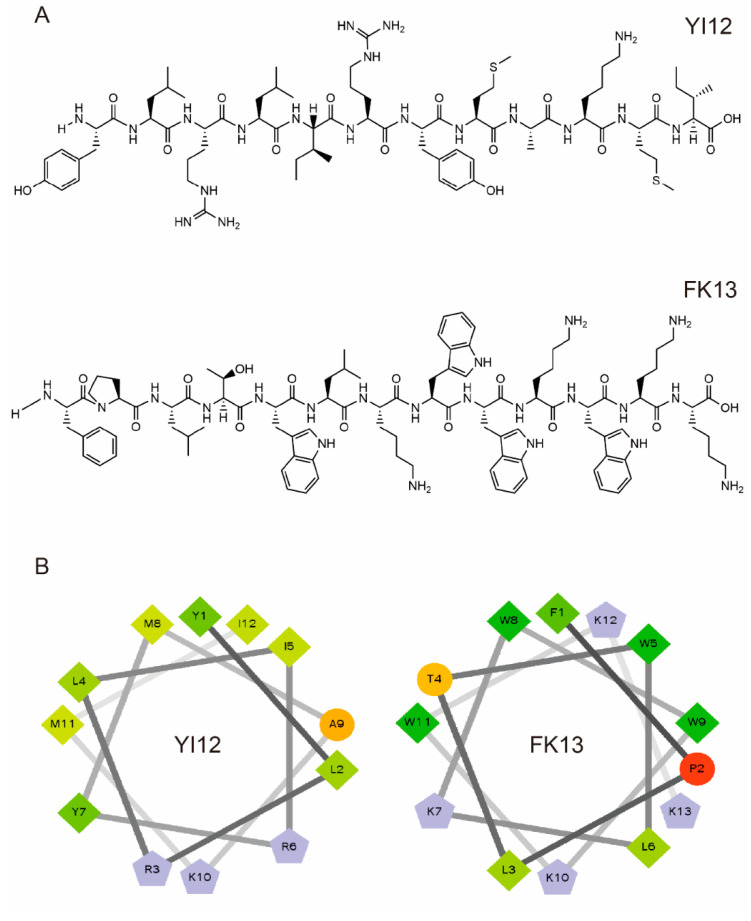
Characterization of YI12 and FK13 peptides. (**A**) Chemical structures of two AMPs. (**B**) Helical wheel projections of YI12 and FK13. Hydrophilic residues—circle shape, hydrophobic residues—diamond shape, potentially positively charged residues—pentagonal shape, potentially negatively charged residues—triangle shape (none). Hydrophobicity is color-coded: the most hydrophobic residues are green, and the amount of green decreases with hydrophobicity. Hydrophilic residues are coded in red, and the amount of red decreases with hydrophilicity. The residues that may be charged are light blue.

We first analyzed the physicochemical properties of two short linear peptides, YI12 and FK13. YI12 comprises 12 amino acids, while FK13 contains 13 amino acids. As illustrated in Table 1, both YI12 and FK13 are cationic polypeptides, possessing net charges of +3 and +4, respectively. Helical wheel projections revealed that hydrophobic residues constitute the majority in both peptides, accompanied by either zero or one hydrophilic residue. The wheel diagram further indicated continuous hydrophobic and cationic faces, as shown in Figure 1. The purity of both peptides surpassed 95%, satisfying the prerequisites for subsequent experimental procedures.

Next, the antibacterial activities of peptide antibiotics YI12 and FK13 against clinically important pathogens, including Gram-positive bacteria (*Staphylococcus aureus*) and Gram-negative bacteria (*Escherichia coli*, *Pseudomonas aeruginosa*, *Acinetobacter baumannii, Klebsiella pneumoniae*), were assessed using standard broth micro-dilution method. YI12 and FK13 exhibited modest antibacterial effects toward Gram-positive bacteria, including MRSA T144, a methicillin-resistant *Staphylococcus aureus* isolate, and *S. aureus* ATCC 29213 with MIC values of 2 μg/mL (YI12) and 4 μg/mL (FK13) (Table 2). Regarding Gram-negative bacteria, the performances of the two AMPs were slightly inferior to that of the Gram-positive bacteria. The results indicated that YI12 and FK13 exhibited a minimum inhibitory concentration (MIC) value of 16 μg/mL against *K. pneumoniae* 700603 while displaying a MIC range of 2 to 8 μg/mL against other various bacterial strains (Table 2). Among these strains, *E. coli* B2 confers multidrug-resistant phenotypes, including colistin and carbapenems. Notably, two peptides showed comparable antibacterial activity against drug-susceptible and -resistant bacteria, indicating that the presence of antibiotic-resistance genes had no effect on the activity of the two peptides.

**Figure 2:**
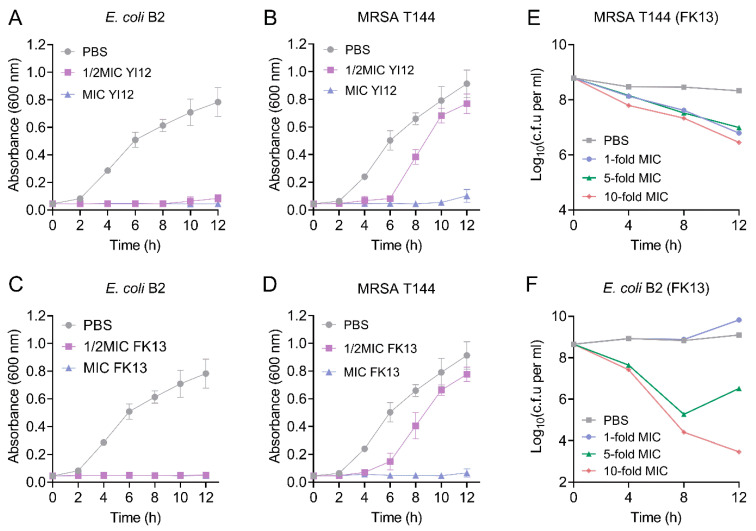
The growth patterns and time-killing curves of the two MDR bacteria exposed to YI12 and FK13, respectively. (**A**–**D**) Growth patterns of MRSA T144 and *E. coli* B2 under YI12 and FK13. (**E**,**F**) Time-dependent killing curves of MRSA T144 and *E. coli* B2 by FK13. Bacteria *isolates* were grown to stationary phase and mixed with 0-, 1-, 5-, and 10-fold MIC of FK13. Data are representative of three independent experiments and shown as mean ± SEM. PBS, phosphate-buffered saline.

Subsequently, we monitored the growth patterns of the two MDR isolates, MRSA T144 and *E. coli* B2, in the presence of YI12 and FK13. As shown in Figure 2, when the concentration of the AMP reached MIC values, a significant inhibitory effect of YI12 and FK13 on the test strain was observed, leading to almost no progress in bacterial growth and reproduction within 12 h. When the concentration of the AMP was at 50% of the MIC, the growth rate of MRSA T144 was completely restricted within the time frame of 0 to 4 h. However, as time passed, the growth of MRSA T144 showed a sharp climb, approaching the drug-free group (Figure 2B,D). By contrast, the growth of *E. coli* B2 was completely restricted in the sub-MICs of the two peptides during 12 h (Figure 2A,C). Based on these findings, we concluded that the two AMPs could effectively prevent the growth of MDR isolates, especially Gram-negative bacteria. Furthermore, we took FK13 as a typical representative to test its bactericidal effect against MRSA T144 and *E. coli* B2. As shown in Figure 2E,F, FK13 exhibited great bactericidal activity against MDR bacteria in a concentration-dependent manner, with both 10-fold MIC concentrations exerting the most obvious CFUs reduction of 2–5 log10 at 12 h.

**Figure 3:**
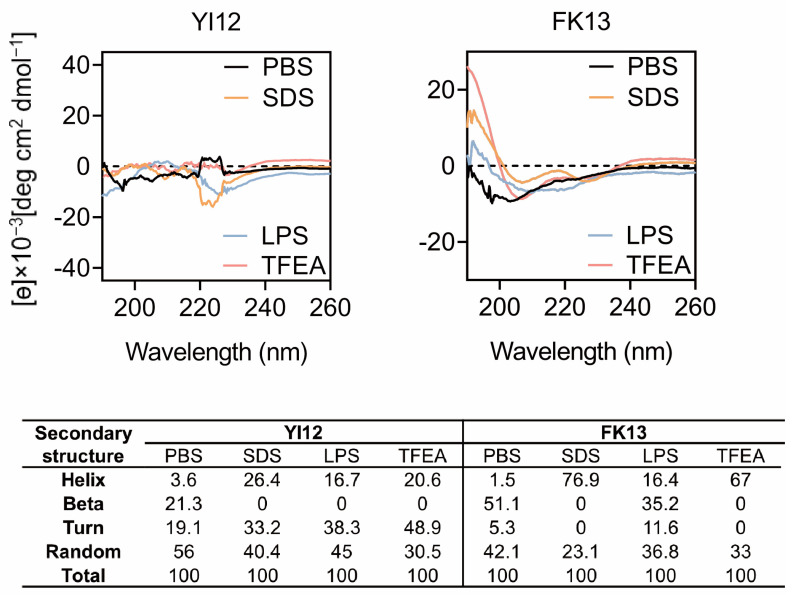
CD spectrum of YI12 and FK13 under different solutions. Use PBS (10 mM, pH = 7.4), LPS (50 µM), SDS (50 mM), and 50% TFEA. Values are averages of three scans of samples with a peptide concentration of 100 μg/mL.

The major reason for AMPs’ bactericidal effects is their unique structures, especially secondary structures influencing peptide-membrane interactions [17]. Therefore, we gained a deep insight into the secondary structures of peptides in various solvents using circular dichroic spectroscopy (CD). Since lipopolysaccharides (LPS) are a crucial constituent of the outer membrane of Gram-negative bacteria [18], we opted to employ LPS to stimulate the bacterial cellular environment. The sodium dodecyl sulfate (SDS) with negative charge was utilized to emulate the anionic membrane environment, while trifluoroethanol (TFEA) was chosen to mimic the hydrophobic environment of the bacterial membrane [19]. Based on the CD spectrum analysis (Figure 3), it was observed that YI12 and FK13 exhibited a combination of helix, turn, and beta-sheet hybrid structures in the PBS solution. Notably, in 50 µM LPS, 50 mM SDS, and 50% TFEA, the beta-sheet and random structures of YI12 disappeared or reduced, respectively, with increasing helix and turn as alternatives. The vanishing beta-sheet and larger proportion of helix also happened in FK13 when it was in 50 mM SDS and TFEA. Meanwhile, the turn ratio was reduced to zero as well, making FK13 become a characteristic α-helix structure in SDS and TFEA environments, evident from the emergence of two distinct negative peaks at 208 nm and 225 nm. These findings suggested that the secondary structures of YI12 and FK13 would present an α-helix conformation upon interactions with bacterial membranes.

### 2.2. Safety and Stability Evaluation of YI12 and FK13

**Figure 4:**
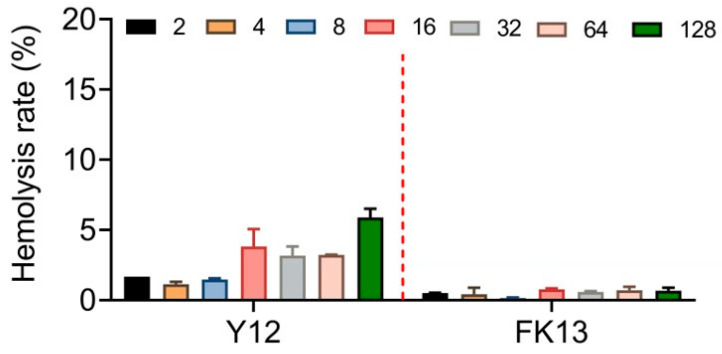
Hemolytic activity evaluation of YI12 and FK13 toward mammalian RBCs. Sterilized PBS served as a negative control, and ddH2O served as a positive control.

The excellent safety and stability of AMPs are their necessary qualifications for efficacy in vivo. Therefore, we first determined the potential cytotoxic effects of YI12 and FK13 by evaluating their hemolysis rates with sheep red blood cells. Generally, FK 13 exhibited lower, even negligible hemolytic rates (≤2%) compared with YI12 (2–6%) in the range of 2–128 μg/mL (Figure 4). Such results indicated that FK13 possessed great safety and biocompatibility, while YI12 had the potential to cause hemolysis at high concentrations.

Stability is also a crucial point of AMPs’ judgment. We first assessed the resistance to heat and acid-base of two peptides by setting up increasing temperatures from 40 °C to 121 °C and different pH conditions ranging from 2 to 12 (Table 3). Delightfully, the two AMPs basically retained their antibacterial activity under different temperatures and pH, with only a two-fold MIC decrease. Notably, the two peptides showed high thermal stability, even at 121 °C conditions for 15 min. Similarly, in the highly alkaline conditions (pH = 12) and acidic conditions (pH = 2), both YI12 and FK13 could maintain their initial potent antibacterial activities against the tested strains. However, when these two AMPs were confronted with ionic environments, especially divalent cations, and hydrolytic enzymes, the situation changed dramatically (Table 3). When exposed to 10 mM monovalent cations like Na^+^ and K^+^, the MIC values were slightly increased, ranging from 2 to 16 μg/mL. But when the peptides were in cationic environments with 10 mM calcium and magnesium ions, the antibacterial activity of the two AMPs was completely abolished, especially for *E. coli* B2. Since Ca^2+^ and Mg^2+^ are stabilizers of the cell membrane, we inferred that YI12 and FK13 might cause cell membrane damage by competing with divalent citations [20,21,22]. Moreover, we also incubated YI12 and FK13 with three types of proteases: pepsin, trypsin, and papain. It turned out that these two peptides were extremely susceptible to protease treatment, with the lowest MIC approaching 64 μg/mL. In the trypsin and papain groups, the MICs were even more than 128 μg/mL, indicating the complete abolishment of the two AMPs’ efficacy. To imitate the internal environment, we further used specific culture media with 10% fetal bovine serum and Dulbecco’s modified eagle medium (DMEM). The results demonstrated that the inhibitory effects of YI12 and FK13 decreased by 2–32 folds.

### 2.3. YI12 and FK13 Alter Membrane Permeability and Dissipate Membrane Potential

**Figure 5:**
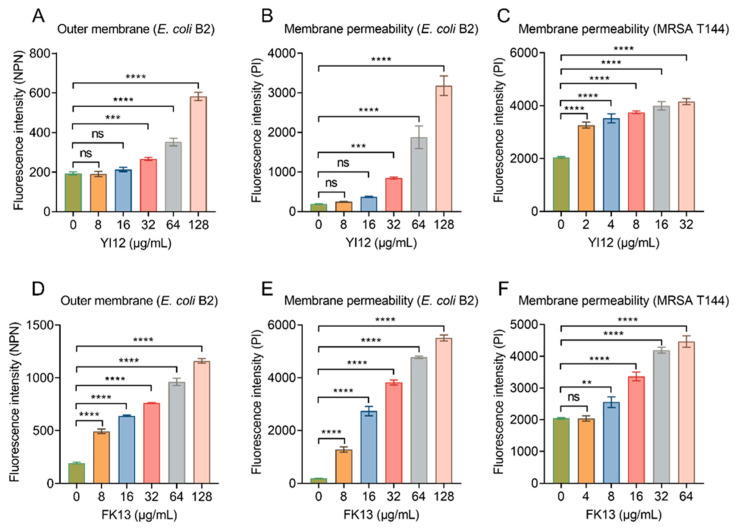
YI12 and FK13 disrupt bacterial membrane permeability. (**A**,**D**) Outer membrane permeability of *E. coli* B2 after exposure to YI12 and FK13, respectively, determined using a fluorescent probe *N*-phenyl-1-naphthylamine (NPN, excitation wavelength 350 nm, emission wavelength 420 nm); (**B**,**C**,**E**,**F**) YI12 and FK13 disrupt bacterial membrane permeability. Detections were performed with the fluorescent probe propidium iodide (PI, excitation 535 nm, emission 615 nm). Statistical analysis was conducted through nonparametric one-way analysis (ns, not significant; ** *p* < 0.01, *** p < 0.001, **** *p* < 0.0001).

After showing the broad-spectrum antibacterial activities of YI12 and FK13, we next tried to explain their potential mechanisms of action. Given their similar physicochemical characteristics and secondary structures, it was postulated that these peptides may share similar antibacterial mechanisms. Considering both peptides were cationic compounds, and divalent cation (outer membrane stabilizers [22]), supplementation would impair their efficacy; we inferred that they exerted a bactericidal effect by targeting the cell membrane. The outer membrane (OM) of Gram-negative bacteria is an important barrier, which normally prevents penetration of hydrophobic fluorescent probe *N*-phenyl-1-naphthylamine (NPN). However, once the OM structures are weakened, NPN can be inserted into the inner leaflet of the OM phospholipids and the cytoplasmic membrane, resulting in increased fluorescence. The results indicated that YI12 could not cause significant changes until its concentrations reached 32 μg/mL. While within the concentration range of 0–128 μg/mL, there was a gradual increase in fluorescent values corresponding to the elevated concentrations of FK13 (Figure 5A,D). Such results suggested that the presence of these two AMPs led to significant impairment of the bacterial OM, with the extent of damage dependent on the AMP concentrations. Next, we evaluated the whole membrane permeability of *E. coli* B2 and MRSA T144 in the presence of the two AMPs using propidium iodide (PI), a membrane-impermeable DNA-binding stain. As shown in Figure 5B, YI12 exhibited similar effects on *E. coli* B2 as the outer membrane’s results: only at concentrations greater than or equal to 32 μg/mL would it have significant influences on membrane permeability. Nevertheless, when YI12 was confronted with Gram-positive bacteria like MRSA T144, it could greatly disrupt membrane permeability (*p* < 0.0001) and presented as a concentration-dependent trend (Figure 5C). FK13 also failed to induce dramatic changes in MRSA T144 when its concentration was less than 8 μg/mL (Figure 5F). But as soon as it encountered Gram-negative bacteria like *E. coli* B2, it could have a significant impact on membrane permeability (Figure 5E). Taken together, these results supported our idea that YI12 and FK13 exhibited bactericidal efficacy by destroying bacterial membrane permeability.

**Figure 6:**
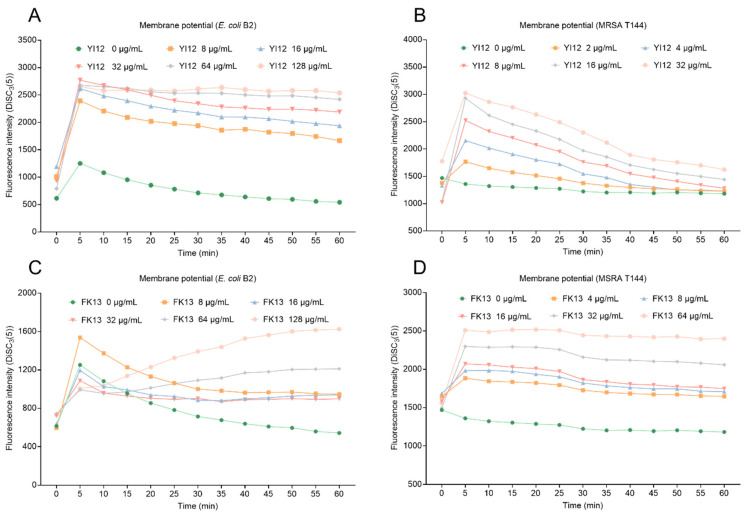
Changes in membrane potential of *E. coli* B2 after exposure to YI12 (**A**) and FK13 (**C**), and MRSA T144 after exposure to YI12 (**B**) and FK13 (**D**). MRSA T144 and *E. coli* B2 membrane potential was determined by monitoring the fluorescence intensity of 3,3′-dipropylthiodicarbocyanine iodine (DiSC_3_(5)). Excitation wavelength 622 nm, emission wavelength 670 nm.

Proton motive force (PMF) is the driving force that facilitates the movement of protons across membranes in the direction of their electrochemical potential gradient [23]. PMF is composed of transmembrane potential (∆Ψ) and transmembrane proton gradient (∆pH). These two elements complement and balance each other to achieve homeostasis. Here, we evaluated the changes in membrane potential in two bacteria exposed to two AMPs using a potentiometric probe DiSC3(5), which would accumulate and manifest with increasing fluorescence once the membrane potential was dissipated. As shown in Figure 6, YI12 and FK13 treatments resulted in increased fluorescence intensity in a concentration-dependent manner in both MRSA T144 and *E. coli* B2, indicating that the two AMPs drastically dissipated the membrane potential of these two bacteria. Collectively, these data demonstrated that YI12 and FK13 could disrupt membrane permeability and dissipate membrane potential.

### 2.4. YI12 and FK13 Trigger the Production of ROS

**Figure 7:**
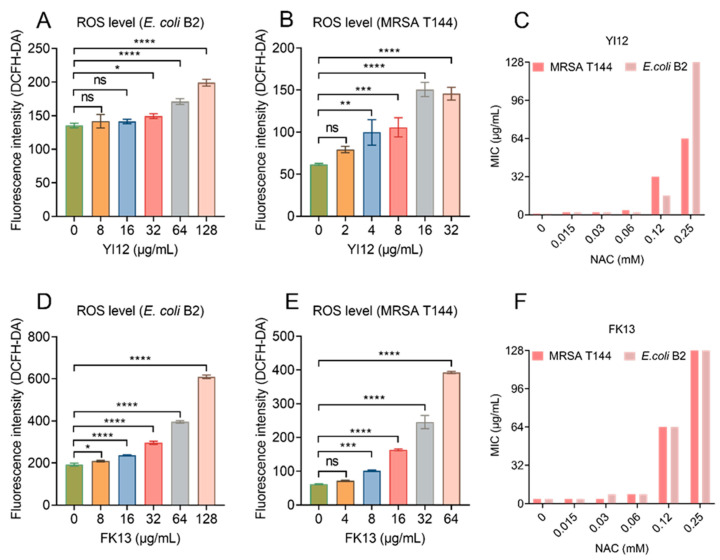
YI12 and FK13 trigger the generation of ROS in *E. coli* B2 and MRSA T144. Generation of ROS in *E. coli* B2 (**A**,**D**) and MRSA T144 (**B**,**E**), determined by 2′,7′-dichlorodihydrofluorescein diacetate (DCFH-DA, excitation wavelength 488 nm, emission wavelength 525 nm). The addition of the ROS scavenger NAC significantly inhibited the effects of YI12 and FK13 on MRSA T144 and *E. coli* B2 (**C**,**F**). Statistical analysis was conducted through nonparametric one-way analysis (ns, not significant; * *p* < 0.05, ** *p* < 0.01, *** p < 0.001, **** *p* < 0.0001).

Previous studies have shown the correlation between membrane depolarization and reactive oxygen species (ROS) production [24], and the over-production of ROS can directly cause bacterial death [25,26]. Therefore, we monitored the ROS level of *E. coli* B2 and MRSA T144 exposed to the two AMPs using 2′,7′-dichlorodihydrofluorescein diacetate (DCFH-DA). The results showed that both peptides induced ROS generation in a dose-dependent manner, with low concentrations causing little or no change in ROS level, while high concentrations could cause a significant increase in fluorescence intensity (Figure 7A,B,D,E). To verify the role of ROS in the antimicrobial activities of YI12 and FK13, we added *N*-acetylcysteine (NAC), a ROS scavenger, in the following MIC value assay. The results showed that the MIC values of the two AMPs were suddenly increased when NAC concentrations achieved 0.12 mM, with a 64-fold increase maximum (Figure 7C,F). Such a phenomenon suggested that the addition of ROS scavengers largely eliminated the antibacterial activities of YI12 and FK13. Hence, it can be concluded that the ROS accumulation elicited by the two AMPs could correspondingly aggravate membrane damage and further disrupt the homeostasis of bacteria.

## 3. Discussion

The emergence of bacteria resistance has led to a progressive decline in the efficacy of antibiotics for treating common infections, and even some diseases caused by certain pathogens tend to be incurable, particularly ESKAPE pathogens (*Enterococcus faecium*, *Staphylococcus aureus*, *Klebsiella pneumoniae*, *Acinetobacter baumannii*, *Pseudomonas aeruginosa*, and *Enterobacter* spp.) [27]. Fortunately, the exceptional diversity and remarkable efficacy exhibited by the AMPs render them uniquely appealing as potential drug candidates amidst the prevailing challenge of drug resistance, with a few already undergoing clinical trials.

The rapid development of computers and various machine algorithms has made de novo design or modification of natural AMPs possible. For instance, in 2018, a peptide termed SAAP-148 was synthesized based on parent peptide LL-37 and has been proven to kill MDR pathogens and destroy biofilms and persister cells, without inducing drug resistance [28]. In addition, a previous study formed a pipeline for the identification and reuse of AMPs from the human gut microbiome by combining diversified neural network models, which is a strong demonstration of the indispensable role of machine learning in AMP development [29].

In this study, we tested the specific performances of YI12 and FK13, which were synthesized by Das et al. with a fully automated computational framework, including physicochemical properties, antibacterial activities, safety assessment, stability evaluation, and their underlying mechanisms. We first confirmed that the two cationic peptides could suppress both Gram-positive or negative MDR isolates, including those carrying *bla*_NDM_ and *mcr*-positive bacteria, with considerably limited MIC values. Moreover, the safety assessment revealed that FK13 had no sign of inducing hemolysis, while YI12 could possibly interrupt mammalian red blood cells’ normal physiological morphology, causing slight hemolysis. We also evaluated two peptides’ stability under various conditions covering high temperatures, acid-base, proteases, and ionic environments. We pointed out that YI12 and FK13 possessed robust resistance to heat and various pH values but were prone to being abolished in the presence of proteases and divalent cations. However, on account of the difference in performances between in vivo and in vitro presented by AMPs, relevant animal tests are required to verify their availability.

Furthermore, we explored their potential mechanisms of action. It was found that both YI12 and FK13 could target the cell membrane, which is also the most important and common mode for most AMPs [30]. Under AMP interactions, the membrane proteins get agglomerated and deactivated, and ion channels are formed, which in turn cause changes in membrane permeability and imbalance between internal and external pressure, finally killing bacteria. Such a process is called the cell membrane damage mechanism [31], including barrel-stave model, toroidal-pore model, and carpet model. In our studies, we found that these two cationic peptides interacted with a negatively charged membrane, by virtue of which it induced significant permeability in the outer and whole cell membrane and exerted antibacterial effects. We also confirmed that YI12 and FK13 could dissipate the membrane potential of PMF, which is a crucial regulator of triphosphate synthesis, molecule transport, and other various biological processes [32]. Further, we uncovered that these two AMPs had the capability to trigger the over-production of ROS and NAC, a ROS scavenger, could eliminate peptides’ bactericidal effects This is consistent with previous reports, which demonstrated that abnormally high ROS levels could disturb the oxidative environment of cells and cause cell death [33].

In summary, our study shows that cationic amphiphilic peptides YI12 and FK13 are able to exhibit bacteriostatic activity, especially against Gram-positive bacteria, which possibly results from their transitions to α-helix conformations upon interactions with bacterial membrane. They also become unstable when confronted with proteases and divalent salt ions. It is worth noting that YI12 has the potential to cause mammalian RBCs lysing at high concentrations while FK13 is safer and more harmless. With regard to mechanisms, both YI12 and FK13 can damage the outer or whole cell membrane, dissipate membrane potential, and induce over-generation of ROS at low concentrations. In future studies, the in vivo therapeutic potential and effectiveness of these two AMPs are warranted to be confirmed.

## 4. Experimental Section

### 4.1. Synthesis and Verification of Antimicrobial Peptides

The antibacterial peptides YI12 and FK13 used in the experiment came from GL Biochem (Shanghai, China) and were both synthesized by peptide solid-phase synthesis (SPPS). Mass spectrometry determined the purified product’s relative molecular mass, which was basically the same as the theoretical molecular weight, indicating that the synthesized antimicrobial peptide was accurate. HPLC results showed that the purity of the peptide was above 95%.

The chemical structure of peptides was drawn using Chem Draw software 20.0. The helical wheel projection and various chemical parameters such as net charge and hydrophobicity were calculated with https://www.donarmstrong.com/cgi-bin/wheel.pl, accessed on 20 July 2024.

### 4.2. Determination of Antibacterial Activity of AMPs

The MIC values of peptide antibiotics YI12 and FK13 were determined via broth microdilution modified by the Clinical and Laboratory Standards Institute (CLSI) [34]. MRSA T144 and *E. coli* B2 were first cultured in Mueller-Hinton broth medium (7.2 ± 0.2 for pH value) for logarithmic growth, and the bacterial solution was diluted 1000 times (3 × 10^8^~9 × 10^8^ CFU/mL) before use. Then, an equal volume (100 μL) of MH broth was placed in a round-bottom polypropylene 96-well plate, diluted the peptide in multiples, and combined with an equal amount of suspension solution. These plates were incubated at 37 °C, for 18~24 h, and the MIC values were defined as the minimum drug concentration for bacterial growth not visible to the naked eye. The experiment was performed with biological replicates.

### 4.3. Time-Dependent Killing

Overnight cultures of MRSA T144 and E. coli B2 were diluted 1:1000 in Mueller Hinton (MH) broth and incubated for 8 h to reach the stationary phase. Subsequently, the bacterial cells were harvested by centrifugation (4000 rpm, 7 min), resuspended in M9 medium, and exposed to 0, 1, 5, and 10 times the MIC of FK13. Bacterial colony counts were performed at 4 h intervals starting from time zero, and the initial colony-forming units per milliliter (CFUs/mL) were calculated.

### 4.4. Secondary Structure Determination

YI12 and FK13 were wholly dissolved in four distinct solvents (0.01 M PBS, pH = 7.2), 50 µM lipopolysaccharide, 50 mM sodium lauryl sulfate, and 50% trifluoroethanol). The resultant circular dichroism spectra were measured under the standard conditions of a bandwidth of 1.0 nm, a step distance of 1 nm, a scanning speed of 100 nm/min, a response time of 8 s, an accumulation amount of 3, and a wavelength range of 196–240 nm at room temperature, using the J-810 circular dichroism spectrometer (Yasko, Japan). Subsequently, the spectra were corrected for background scatter by subtracting that of the buffer.

### 4.5. Analysis of Hemolytic Activity

Fresh sheep erythrocytes were centrifuged at 3000 g for 10 min at 4 °C and suspended in phosphate buffer solution (0.01 M, pH = 7.4) to obtain an erythrocyte suspension with a dilution of 8%. Then, it was mixed with the pre-configured AMP solutions whose concentration increased in a gradient, and the mixture was placed at 37 °C for 1 h. After the incubation, the supernatant was taken out, centrifuged at 3000× *g* for 5 min, and then transferred to a flat-bottomed 96-well plate. The released amount of hemoglobin (OD_576_) was measured with a microplate reader (Tecan, Männedorf, Switzerland), according to which the corresponding hemolysis rate was calculated. Hemolysis rate (%) = [(OD_570_ sample absorbance − OD_570_ negative control absorbance)/(OD_570_ positive control absorbance − OD_570_ negative control absorbance)] × 100%. Negative and positive control were set as sterilized PBS and double distilled water (ddH_2_O), respectively. The 2% hemolytic rate was defined as the dividing line between high and low hemolytic activity [35].

### 4.6. Physiological Stability Determination of AMPs

#### 4.6.1. Salt Ion and Serum Stability

NaCl, KCl, MgCl_2_, CaCl_2_ solutions, 10% Dulbecco’s Modified Eagle medium (DMEM), and fetal bovine serum (FBS) were added to the diluted bacterial solution to prepare a saline-ion bacterial mixture with a final concentration of 10 mM, and the corresponding MICs were determined.

#### 4.6.2. pH and Temperature Stability

Different concentrations of YI12 and FK13 solutions were aliquoted in EP tubes, incubated at different pH (2, 4, 6, 8, 10, and 12) for 1 h, and then the pH values were adjusted to the initial pH with hydrochloric acid and sodium hydroxide. MIC was determined to evaluate the acidic and alkaline sensitivity of the AMPs. To assess the thermal stability, different concentrations of AMPs were placed in a thermostatic pot to varying temperatures for 1 h, and then the MIC of these samples was detected.

#### 4.6.3. Protease Stability

The AMPs were mixed with pepsin, trypsin, and papain solution to prepare a final concentration of 10 mM sample solution, incubated at 37 °C for 1 h. Then, the samples were fully made, precipitated with acetonitrile to prevent further degradation, and centrifuged at 3000 r/min. The residual antibacterial activities of the samples were detected by determining the changes in MIC values.

#### 4.6.4. Detection of Outer Membrane Integrity

The outer membrane permeability of bacteria treated with YI12 or FK13 was assessed using the lipophilic dye NPN probe. MRSA T144 and *E. coli* B2 were cultured to the logarithmic growth phase and adjusted to an OD_600_ of 0.5. Then, a final concentration of 0.1 μM NPN was added and incubated at 37 °C for 30 min at 200 rpm. After incubation with increasing concentrations of AMPs in a black 96-well flat-bottom plate for 1 h, fluorescence intensities were measured at an excitation wavelength of 350 nm and an emission wavelength of 420 nm.

#### 4.6.5. Detection of Cell Membrane Permeability

Overnight cultured MRSA T144 and *E. coli* B2 were diluted into MH broth at a dilution ratio of 1:100, placed on a shaker at 37 °C and 200 rpm for 4 h, and then the cells were collected and washed with PBS (pH = 7.4), with resuspension to an OD_600_ of 0.5. A DNA-binding dye propidium iodide (final concentration, 0.5 µM) was added and incubated at 37 °C for 30 min in the dark. After incubating with increasing concentrations of AMPs in a 37 °C incubator for 1 h, the fluorescence intensity at an excitation wavelength of 535 nm and an emission wavelength of 615 nm was measured using a microplate reader (Tecan).

#### 4.6.6. Membrane Potential Detection

The transmembrane potential of the bacterial plasma membrane was determined using a probe 3,3′-dipropylthiadicarbocyanine iodine (DiSC_3_(5)). MRSA T144 and *E. coli* B2 cultured overnight were diluted in a blank MH broth and cultured at 37 °C in a 200 rpm shaking table for 4 h. The bacteria were collected, washed with PBS, and suspended in OD_600_ of 0.5. DiSC_3_(5) (final concentration, 0.5 µM) was added and incubated at 37 °C without light for 30 min. Bacterial cells labeled with 190 μL probe were spread on a sterile black 96-well flat plate along with the antibacterial peptide solution with 10 μL increasing concentration of 0–128 μg/mL. After incubation at 37 °C for 1 h, the fluorescence intensity was measured by a microplate reader (Tecan), and the excitation and emission wavelengths were set to 622 nm and 670 nm, respectively, with a time interval of 5 min.

#### 4.6.7. Reactive Oxygen Species (ROS) Measurement

The effects of YI12 and KF13 acting on ROS in MRSA T144 and *E. coli* B2 were evaluated with fluorescent probe 2′,7′-dichlorodihydrofluorescein diacetate (DCFH-DA) (Beyotime). DCFH-DA (10 µM) was pre-incubated with bacterial cells for 30 min, then centrifuged and washed with PBS to remove excess fluorescent probes that did not enter the cells. After incubating with different concentrations of AMPs (0, 8, 64, and 128 μg/mL) for 1 h, the fluorescence intensity at an excitation wavelength of 488 nm and an emission wavelength of 525 nm was measured.

### 4.7. Statistical Analysis

Data were presented as mean ± standard deviation (SD) and analyzed using GraphPad software (GraphPad Prism Version 8.0 for Windows). Statistical analysis was conducted through an unpaired *t*-test between two groups or a nonparametric one-way analysis of variance among multiple groups (ns, not significant; * *p* < 0.05, ** *p* < 0.01, *** *p* < 0.001, **** *p* < 0.0001).

## Figures and Tables

**Table 1:** Physicochemical properties of YI12 and FK13. ^#^ The isoelectric points (pI) of peptides were predicted by ExPASy (https://web.expasy.org/protparam/), accessed on 20 July 2024.

Name	Sequence (N→C)	Formula	MW	Net Charge	pI ^#^	Purity (%)
YI12	YLRLIRYMAKMI-NH_2_	C_73_H_123_N_19_O_15_S_2_	1571.72	+3	10.28	96.73%
FK13	FPLTWLKWWKWKK-NH_2_	C_98_H_135_N_21_O_15_	1847.28	+4	10.48	95.23%

**Table 2:** Antibacterial spectrum of YI12 and FK13 (MIC, μg/mL). MRSA, methicillin-resistant *Staphylococcus aureus*. G^+^, Gram-positive bacteria; G^−^, Gram-negative bacteria.

Organisms	Phenotypes	YI12	FK13
*S. aureus* ATCC 29213	G^+^, sensitive	2	4
MRSA T144	G^+^, methicillin-resistant	2	2–4
*E*. *coli* ATCC 25922	G^−^, sensitive	8	8
*E*. *coli* MG1655	G^−^, sensitive	2	4
*E*. *coli* B2 (*mcr-1* + *bla*_NDM-5_)	G^−^, colistin/carbapenem-resistant	4	4–8
*P. aeruginosa* PA14	G^−^, ampicillin-resistant	8	2
*S. enteritidis* ATCC 13076	G^−^, sensitive	8	4
*A. baumannii* ATCC 19606	G^−^, sensitive	2	4
*K. pneumoniae* ATCC 700603	G^−^, sensitive	16	16

**Table 3:** Stability evaluation of antimicrobial peptides YI12 and FK13 against MRSA T144 and *E. coli* B2 (MIC, μg/mL).

Treatments	YI12		FK13	
	MRSA T144	*E. coli* B2	MRSA T144	*E. coli* B2
**Control**	2	4	2	4
**Temperature**				
40 °C	4	4	2	8
60 °C	4	8	2	8
80 °C	4	8	2	8
100 °C	4	4	2	8
121 °C	4	4	2	4
**pH**				
2	2	8	4	4
4	2	4	4	4
6	2	4	2	4
8	2	4	2	4
10	2	8	2	4
12	4	8	2	4
**Salts (10 mM)**				
NaCl	2	16	4	4
KCl	8	4	16	4
MgCl_2_	4	>128	16	>128
CaCl_2_	>128	>128	>128	>128
**Protease (1 mg/mL)**				
Pepsin	64	64	64	64
Trypsin	64	>128	64	>128
Papain	>128	>128	>128	>128
**Serum (10%)**	4	32	16	32
**DMEM (10%)**	64	16	8	16

## Data Availability

Data are contained within the article.
